# Editorial: Character, responsibility, and well-being: influences on mental health and constructive behavior patterns

**DOI:** 10.3389/fpsyg.2015.01079

**Published:** 2015-07-29

**Authors:** Danilo Garcia, Ann-Christine Andersson Arntén, Trevor Archer

**Affiliations:** ^1^Network for Empowerment and Well-BeingGothenburg, Sweden; ^2^Centre for Ethics, Law and Mental Health, Institute of Neuroscience and Physiology, University of GothenburgGothenburg, Sweden; ^3^Department of Psychology, University of GothenburgGothenburg, Sweden

**Keywords:** character, personality, well-being, responsibility, agency, communion, spirituality

Character can be defined as self-aware knowledge that helps the individual to set goals, values, and ethical principles (Cloninger, 2004). This meta-cognitive dimension of human personality involves “Theory of Mind,” and is positively related to measures of well-being, mental health, and constructive behavior patterns. Research from at least three different fields, cultural (Shweder et al., 1997), personality (Cloninger, 2004), and social psychology (Abele and Wojciszke, 2007) suggest that character can be organized along three broad principles: agency, which is related to the autonomy and the fulfillment and enhancement of the self; communion, which is related to engagement in the protection and relations to others such as families, companies or nations; and spirituality, which is related to the human ability to transcend the self and find and interconnection with all life and appreciation of the whole world around us (Haidt, 2006; Cloninger, 2013).

Using the Temperament and Character Inventory (Cloninger et al., 1993) researchers have found that Self-directedness (i.e., agency), Cooperativeness (i.e., communion), and Self-transcendence (i.e., Spirituality) are associated to high levels of happiness, psychological well-being, and less violent behavior (Garcia et al., 2013, 2015; Nima and Garcia, 2015; Mousavi et al., 2015). Moreover, low Self-directedness and Cooperativeness is recurrent among individuals with all types of mental health problems, such as, depression, schizophrenia, anxiety disorder, autism spectrum disorders, attention deficit/hyperactivity disorder, and etcetera. Self-transcendence, in coherence with Self-directedness and Cooperativeness, guides the individual to seek self-realization in harmony with others and nature in the changing world (Cloninger, 2013). Seeing character as self-awareness of the self in three dimensions has also been associated to human responsibility and empowerment (Nima et al., 2012; Schütz et al., 2013a,b; Cloninger and Garcia, 2015).

In this Research Topic researchers offer their perspective on character, responsibility, and well-being. Ruch and his colleagues, using other measures for character, offer a series of articles ranging from life satisfaction among religious people (Berthold and Ruch, 2014) to good character in school (Wagner and Ruch, 2015). Abele develops the idea of how communal values need to be pursued in agentic ways (Abele, 2014), while Garcia and his colleagues give an insight into the possible use of character-centered teams at work places (e.g., Garcia et al., 2014a) and also its etiology in adolescence (Garcia et al., 2014b). Continuing this line, Jeppsson (2014) gives a philosophical perspective on responsibility in the field of criminal justice. Finally, Moreira et al. (2015) show the importance of character and its relation to well-being during adolescence, while Nilsson (2014) gives a critical opinion of the need of introducing the perspective of worldview when studying the association between personality and well-being.

With this range of different takes on the interactions between character, responsibility and well-being we hope to give a new perspective on the investigation of personality's role on human health and well-being.

## Conflict of interest statement

The authors declare that the research was conducted in the absence of any commercial or financial relationships that could be construed as a potential conflict of interest.

## References

[B1] Abele A. E. (2014). Pursuit of communal values in an agentic manner: a way to happiness? Front. Psychol. 5:1320. 10.3389/fpsyg.2014.01320 25477843 PMC4235276

[B2] Abele A. E. Wojciszke B. (2007). Agency and communion from the perspective of self versus others. J. Pers. Soc. Psychol. 93, 751–763. 10.1037/0022-3514.93.5.751 17983298

[B3] Berthold A. Ruch W. (2014). Satisfaction with life and character strengths of non-religious and religious people: it's practicing one's religion that makes the difference. Front. Psychol. 5:876. 10.3389/fpsyg.2014.00876 25177303 PMC4132480

[B4] Cloninger C. R. (2004). Feeling Good: The Science of Well-being. New York, NY: Oxford University Press.

[B5] Cloninger C. R. (2013). What makes people healthy, happy, and fulfilled in the face of current world challenges? Mens Sana Monogr. 1, 16–24. 10.4103/0973-1229.109288 23678235 PMC3653221

[B6] Cloninger C. R. Garcia D. (2015). The heritability and development of positive affect and emotionality, in Genetics of Psychological Well-Being - The Role of Heritability and Genetics in Positive Psychology, ed Pluess M. (New York, NY: Oxford University Press), 97–113.

[B7] Cloninger C. R. Svrakic D. M. Przybeck T. R. (1993). A Psychobiological model of temperament and character. Arch. Gen. Psychiatry 50, 975–989. 8250684 10.1001/archpsyc.1993.01820240059008

[B8] Garcia D. Ghiabi B. Rosenberg P. Nima A. A. Archer T. (2015). Differences between affective profiles in temperament and character in salvadorians: the self-fulfilling experience as a function of agentic (Self-directedness) and communal (Cooperativeness) values. Int. J. Happiness Dev. 2, 22–37. 10.1504/IJHD.2015.067592

[B9] Garcia D. Lindskär E. Archer T. (2014a). To schedule or not to schedule? Agentic and cooperative teams at call centers. Front. Psychol. 5:999 10.3389/fpsyg.2014.00999 PMC4165209 25278912

[B10] Garcia D. Nima A. A. Archer T. (2013). International note: temperament and character's relationship to subjective well-being in salvadorian adolescents and young adults. J. Adolesc. 36, 1115–1119. 10.1016/j.adolescence.2013.08.018 24215958

[B11] Garcia D. Stråge A. Lundström S. Radovic S. Brändström S. Råstam M. (2014b). Responsibility and cooperativeness are constrained, not determined. Front. Psychol. 5:308 10.3389/fpsyg.2014.00308 PMC4019847 24847287

[B12] Haidt J. (2006). The Happiness Hypothesis: Finding Modern Truth in Ancient Wisdom. New York, NY: Basic Books.

[B13] Jeppsson S. M. I. (2014). Responsibility problems for criminal justice. Front. Psychol. 5:821. 10.3389/fpsyg.2014.00821 25120520 PMC4114194

[B14] Moreira P. A. S. Cloninger C. R. Dinis L. Sá L. Oliveira J. T. Dias A. (2015). Personality and well-being in adolescents. Front. Psychol. 5:1494 10.3389/fpsyg.2014.01494 PMC4285735 25610408

[B15] Mousavi F. Rozsa S. Nilsson T. Archer T. Anckarsäter H. Garcia D. (2015). Persistence, not self-directedness, cooperativeness or self-transcendence, is related to twins' cognitive abilities. (In press). 10.7717/peerj.1195 PMC4548492 26312186

[B16] Nilsson A. (2014). A non-reductive science of personality, character, and well-being must take the person's worldview into account. Front. Psychol. 5:961. 10.3389/fpsyg.2014.00961 25221538 PMC4147830

[B17] Nima A. A. Archer T. Garcia D. (2012). Adolescents' happiness-increasing strategies, temperament, and character: mediation models on subjective well-being. Health 4, 802–810. 10.4236/health.2012.410124

[B18] Nima A. A. Garcia D. (2015). Factor structure of the Happiness-Increasing Strategies Scales (H-ISS): activities and coping strategies in relation to positive and negative affect. PeerJ 3:e1059. 10.7717/peerj.1059 26157626 PMC4493682

[B19] Schütz E. Archer T. Garcia D. (2013a). Character profiles and adolescents' self-reported affect. Pers. Individ. Diff. 54, 841–844. 10.1016/j.paid.2012.12.020

[B20] Schütz E. Sailer U. Nima A. Rosenberg P. Andersson Arntén A.-C. Archer T.. (2013b). The affective profiles in the USA: happiness, depression, life satisfaction, and happiness- increasing strategies. Peer J. 1:e156. 10.7717/peerj.156 24058884 PMC3775633

[B21] Shweder R. A. Much N. C. Mahapatra M. Park L. (1997). The ‘Big Three’ of morality (autonomy, community, divinity) and the ‘Big Three’ explanations of suffering, in Morality and Health, eds Brandt M. Rozin P. (New York, NY: Routledge), 119–172.

[B22] Wagner L. Ruch W. (2015). Good character at school: positive classroom behavior mediates the link between character strengths and school achievement. Front. Psychol. 6:610. 10.3389/fpsyg.2015.00610 26029144 PMC4432234

